# Seasonal Changes in Testes Vascularisation in the Domestic Cat (*Felis domesticus*): Evaluation of Microvasculature, Angiogenic Activity, and Endothelial Cell Expression

**DOI:** 10.1155/2012/583798

**Published:** 2012-02-08

**Authors:** Graça Alexandre-Pires, Luísa Mateus, Catarina Martins, Graça Ferreira-Dias

**Affiliations:** ^1^Morphology and Function Department, CIISA, Faculty of Veterinary Medicine of Lisbon, Technical University of Lisbon, 1300-477 Lisbon, Portugal; ^2^Clinical Department, CIISA, Faculty of Veterinary Medicine of Lisbon, 1300-477 Lisbon, Portugal; ^3^Immunology Department, Faculty of Medical Sciences, 1169-056 Lisbon, Portugal

## Abstract

Some male seasonal breeders undergo testicular growth and regression throughout the year. The objective of this study was to understand the effect of seasonality on: (i) microvasculature of cat testes; (ii) angiogenic activity in testicular tissue *in vitro*; and (iii) testicular endothelial cells expression throughout the year. Testicular vascular areas increased in March and April, June and July, being the highest in November and December. Testes tissue differently stimulated *in vitro* angiogenic activity, according to seasonality, being more evident in February, and November and December. Even though CD143 expression was higher in December, smaller peaks were present in April and July. As changes in angiogenesis may play a role on testes vascular growth and regression during the breeding and non-breeding seasons, data suggest that testicular vascularisation in cats is increased in three photoperiod windows of time, November/December, March/April and June/July. This increase in testicular vascularisation might be related to higher seasonal sexual activity in cats, which is in agreement with the fact that most queens give birth at the beginning of the year, between May and July, and in September.

## 1. Introduction

The study of the vasculature of the testis has attracted scientists' attention for many centuries, especially at the end of the 19th and throughout the 20th century. This research has been performed in a large variety of animal species such as rat, mouse, rabbit, guinea pig, dog, ram, bull, boar, horse, marsupials, man, and other primates [1–4]. It has been long known that the physiologic role of the pampiniform plexus, on thermoregulation of the testes. This is a highly efficient countercurrent heat exchanger in which the arterial blood is precooled before it reaches the testis, while venous blood is warmed to body temperature before it returns to the abdomen [5]. It is nevertheless very rare to find references on the vasculature of cat testes (*Felis domesticus*).

Angiogenesis is physiologically modulated through a dynamic balance between the production and release of angiogenic/mitogenic growth factors or antiangiogenic/anti-mitogenic substances [6–9]. In the adult, physiological angiogenesis is mostly restricted to the female reproductive tract during the ovarian/uterine cycle [10–13]. Nevertheless, in the male, physiologic gonadal angiogenesis has also been described. Testicular angiogenesis is known to increase during testicular recrudescence in seasonal breeders such as the hamster [14] or to decrease in response to food restriction in rabbits [15].

Testis mass and sperm production vary throughout the year in animals such as the bonnet monkey or the European hamster [16–18]. It is also known that sexual activity is influenced by geographic location [19, 20]. Seasonality of domestic feral cats in the northern hemisphere is referred to occur throughout a wide range of months, when day light is prolonged for 12 hours or longer [21]. Testicular growth and regression occur throughout the year with simultaneous fast changes in blood flow that appears to be stimulated by angiogenic substances and inhibited by antiangiogenic factors [22–25]. The objective of this study was to understand the effect of seasonality on (i) microvasculature of cat testes; (ii) angiogenic activity in testicular tissue *in vitro*; (iii) testicular endothelial cells expression throughout the year (January–December).

## 2. Material and Methods

### 2.1. Sample Collection

A total of 516 testes from adult domestic stray cats (*n* = 378) were collected and used for the present work. Testes were obtained from cats of indeterminate breed, weight, and age that underwent euthanasia, at the Official Kennel of Lisbon, Portugal (latitude 38°43′N). This organization is recognized by the European Convention for the Protection of Small Animals.

 Experiments were monitored by competent veterinary authorities and approved by the ethical committee of the Faculty of Veterinary Medicine (Lisbon, Portugal). Authors G. Alexandre-Pires and L. Mateus, are holders of an FELASA (Federation of European Laboratory Animal Science Associations) grade C certificate, which allows designing and conducting laboratory animal experimentation in the European Union.

### 2.2. Injection-Diaphanisation Technique

Soon after euthanasia, 10 testis were collected from adult cats (*n* = 5) in order to be used for injection-diaphanisation technique. injection-diaphaniszation or clearing technique was performed as follows: (i) washing of the vascular bed with distilled water: (ii) injection of 10% jelly and a colloidal suspension of barium sulphate (Micropaque-Laboratório Guerbet, Portugal), in a 25% dilution, at 37°C; (iii) fixation phase which was performed by using 10% formaldehyde solution; (iv) bleaching phase, that was accomplished by immersion of testis in hydrogen peroxide for 30 minutes. (v) “Freeze substitution method” was performed, which consists in freezing the organ to −20°C and damping it in pure commercial acetone at −20°C (Maialab, Portugal). (vi) Clarification phase was followed, which consists of the immersion of testis' slices in Spalteholz liquid, composed of benzyl benzoate and methyl salicilate (ChemNet Europe). Finally, samples were observed by transillumination (Wild, M3Z, Switzerland).

### 2.3. Scanning Electron Microscopy

Scanning electron microscopy (SEM) was performed on microvascular corrosion casts of cat testicles (*n* = 10) and intact testicular tissue (*n* = 5). SEM technique for microvascular corrosion casts was performed according to the following protocol: immediately after euthanasia a polyethylene catheter was introduced in the thoracic aorta of the cat. *Postmortem* fixation was achieved by injection of 2.2% glutaraldehyde in phosphate buffer solution (pH 7.28) inside the vascular bed. After, an acrylic resin known as “Mercox” (Vilene Hospital, Tokyo, Japan) was injected through the catheter. After polymerization of the resin the corrosion was carried out through the use of a 24% NaOH solution (SIGMA, Portugal). Testis corrosion casts were mounted on stubs, coated with gold palladium and observed in a scanning electronic microscope (JEOL-JSM-5410).

Scanning electronic microscopy technique for intact tissue was performed following the next steps: cat testes were immersed in Karnovsky's solution (Sigma-Aldrich, Portugal). Afterwards they were rinsed in cacodylate buffer and post-fixed in a 2% osmium tetroxide solution for 1 hour. The pieces were rinsed once again with cacodylate buffer and subsequently dehydrated in a graded ethanol series. Samples were dried using the critical point drying method and coated with gold salts. The same electronic microscope (JEOL-JSM-5410) was used to study and photograph these structures.

### 2.4. Flow Cytometry Analysis

For one year, a total of 236 cat testes were collected monthly immediately after euthanasia (*n* = 118). Testes were removed with a surgical blade and collected into a sterile tube with 1 mL of RPMI 1640 (Gibco-Brl, USA). After disaggregation of the tissue with a surgical blade samples that corresponded to the total amount of testis mass were centrifuged at 190 ×g for 10 min and adjusted to a concentration of 10^6^ cells per 100 *μ*L in phosphate-buffered saline solution (PBS). Aliquots of 10^6^ cells were incubated for 30 min at 4°C with 10 *μ*L of CD143 mouse anti-human antibody (MCA2057 from AbdSerotec, 170 KD somatic form), which recognizes angiotensin converting enzyme mainly in endothelial cells. Red blood cells were lysed with BD FACS Lysing Solution (BD Biosciences, San Jose, CA, USA) for 10 minutes. After centrifugation, at 190 g for 10 minutes, the pellet was resuspended in 500 *μ*L of BD FACS Flow (BD Biosciences, San Jose, CA, USA). Fluorochrome-conjugated secondary antibodies, namely Goat anti-mouse IgG : RPE, (from AbDSerotec, UK) were added, vortexed and incubated for 15–30 minutes. Finally, to wash off antibody excess following staining, 1.5 mL of PBS was added to each tube. Cell acquisition was performed on a BD FACS Calibur flow cytometer (BD Biosciences, San Jose, CA, USA) and data were analyzed using Paint-A-Gate Pro and Cell-Quest Pro software (BD Biosciences, San Jose, CA, USA).

### 2.5. Microvascular Density Evaluation

Monthly and throughout a whole year, testes were collected after euthanasia from domestic stray cats, for histology and for tissue culture (*n* = 120, from the total number of animals) were collected. After removal of the epididymis, testes were cut transversally for different assays such as the determination of vascular areas and mitogenesis assays.

For histological studies, samples were placed in 4% buffered formaldehyde, fixed overnight, and dehydrated in a series of ethanol solutions and embedded in paraffin. Sections were cut with a rotary microtome (Leica Microsystems Nussloch GmbH, Nussloch, Germany), stained with Periodic Schiff reagent (Sigma), and evaluated under a light microscope (Olympus Ck40, Wetzlar-Nauborn, Germany). For each testis, 28 randomly selected fields were photographed using a light microscope at 1000x magnification. Testicular vascular areas were measured by using computerized image analysis (Scion Image, NIH, USA). Vascular density (arterioles, venules, and capillaries) was assessed as the percentage of the area occupied by blood vessels with respect to the entire area of each micrograph [12, 15, 26]. For each animal/testis, the vascular area was considered as the mean value of the area of blood vessels for each animal on the same 28 randomly selected fields.

### 2.6. Mitogenesis Assays

For mitogenesis assays, 60 mg of testicular tissue (10 cats from each month) were incubated in 2 mL culture medium, for 18 hours, in a tissue incubator (Biosafe Eco-Integra Biosciences, Chur, Switzerland) at 37°C, 5% CO_2_, 95% air, on a shaker (Titertek, Huntsville, AL, USA) AT 1500 RPM. Culture medium consisted of DMEM and Ham's F12 (1 : 1 V/V) supplemented with 0.1 BSA, penicillin (100 IU/mL), and streptomycin (100 *μ*g/mL), being all reagents from Sigma. After incubation, media were stored at −70°C for further mitogenesis assays. Negative controls consisted of culture medium alone.

The ability of media conditioned by testicular tissue to stimulate angiogenic activity was indirectly assessed by mitogenesis/proliferation of Bovine Aortic Endothelial Cells (BAEC; kindly donated by Dr. Dale A. Redmer, North Dakota State University, Fargo, ND, USA), as referred by Redmer et al., [27].

Briefly, BAEC cells (2 × 10^4^ cells/mL) were allowed to attach to the bottom of 24-well culture plate (Nucleon-Nunc, Ballerup, Denmark) for 24 hours, in a tissue incubator (Biosafe Eco-Integra Biosciences) at 37°C, 5% CO_2_, 95% air [12]. Samples of testicular conditioned media (or controls) were added in triplicate wells at a final concentration of 30% (30% conditioned media + 70% DMEM) and incubated for 72 hours. Vascular endothelial growth factor (VEGF; Sigma) was used as a positive control, at different concentrations (10, 20, 25 and 50 ng/mL) to evaluate the ability of an angiogenic factor to stimulate BAEC proliferation under the same experimental conditions [12]. In order to assess BAEC proliferative response, the number of these cells in each well was determined using a Neubauer chamber under the light microscope (Olympus CK40) and further compared with positive controls (VEGF) and negative controls (no tissue added). The percentage of BAEC proliferation in media conditioned by testes was calculated with respect to negative controls. Cell proliferation or mitogenesis in response to negative control culture media was considered 100%.

### 2.7. Statistical Analysis

Statistical significance among data regarding microvascular areas and angiogenic activity in cat testes throughout the year were analysed by one-way ANOVA. The level of significance was set at *P* < 0.05. Whenever a significant difference was observed, a Bonferroni multiple-comparison test was performed.

## 3. Results

### 3.1. Testicular Microvascularization

**Figure 1:**
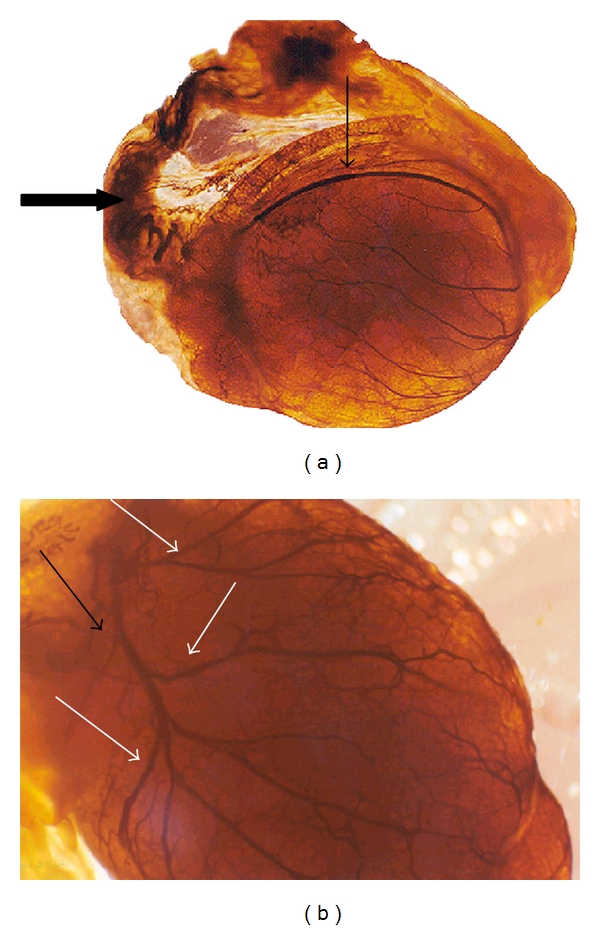
(a and b) Injection-diaphanisation technique: a single testicular artery, slightly convoluted in the spermatic cord (thick arrow). This vessel winds around its caudal pole to reach the ventral border (thin arrow), where it starts to branch in a monopodical way, giving away branches to both sides of the testis (white arrows).

**Figure 2:**
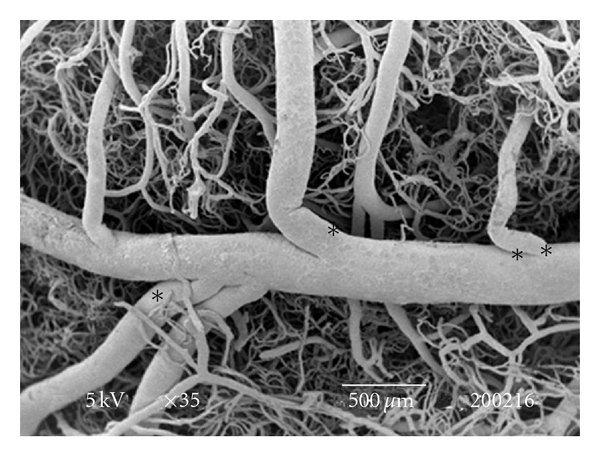
SEM: micrograph of cat testicular artery that gives away branches to both sides of the testis. These vessels are commonly the 1st- (*) and 2nd-order arteries (**). Bar = 500 *μ*m.

**Figure 3:**
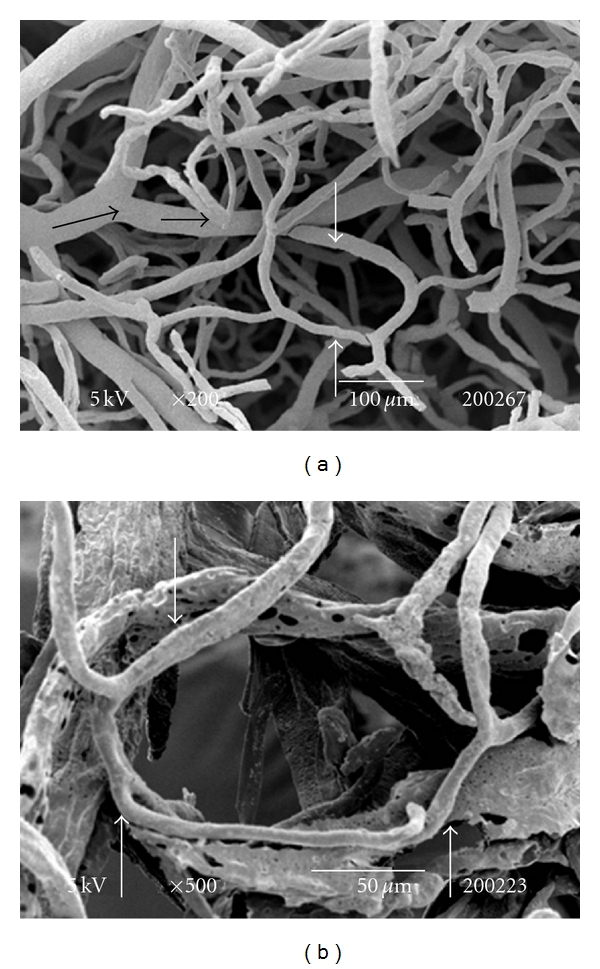
(a) SEM: dichotomic branches from different pre-capillary arterioles (black arrow) show peritubular patterns determined by the layout of the seminiferous tubules (white arrow). Bar = 100 *μ*m. (b) SEM: Idem. Peritubular vascularisation (arrows). Bar = 50 *μ*m.

**Figure 4:**
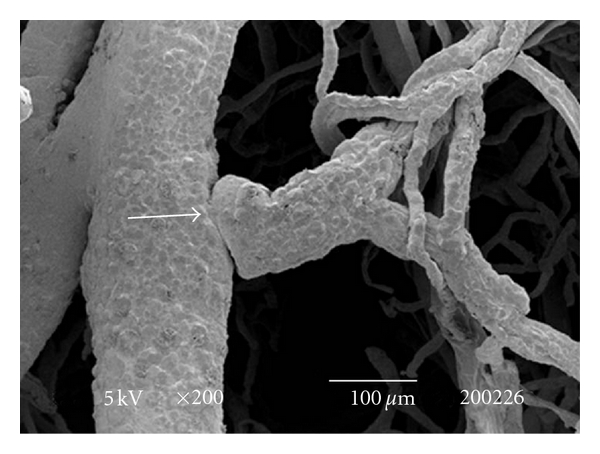
SEM: an ostial valvulae (arrow) can be observed in a 2nd order artery. Bar = 100 *μ*m.

**Figure 5:**
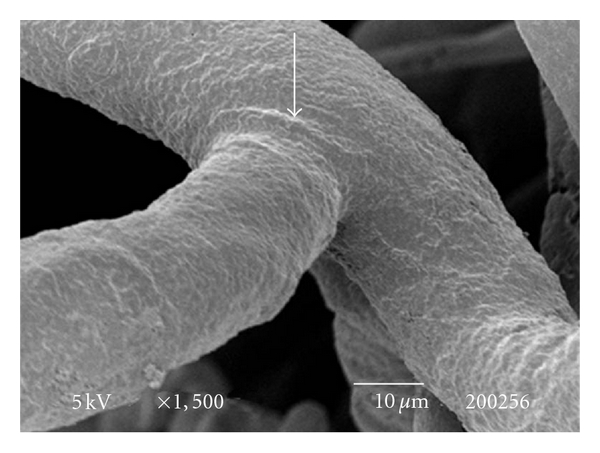
SEM: intra-arterial cushions are observed in pre-capillary arterioles (arrow). Bar = 10 *μ*m.

The testes microvascularization was classified on the basis of the classification adopted and applied to the placenta and to the dog's spleen and third eyelid [28–30]. The present study has shown that cat testis is supplied by a single testicular artery, slightly convoluted in the spermatic cord, where it is surrounded by largely anastomosed veins of the very rich pampiniform plexus (Figure 1(a)). Upon reaching the hilum of the testis, the testicular artery winds around its caudal pole to reach the ventral border, where it starts to branch in a monopodical way, giving away branches to both sides of the testis (Figures 1(b) and 2). These branches, usually small calibre arteries or 1st-order arterioles, course undivided for a variable length under the tunica albuginea and then sink into the testicular parenchyma where they branch, originating successively 1st-order (200–100 *μ*m), 2nd-order (100–30 *μ*m), and pre-capillary or terminal arterioles (30–10 *μ*m). Although first divisions of the testicular artery are given in a monopodical way, branches located distally from the artery originate branches in a dichotomic way and the same pattern can be seen inside the testicular parenchyma (Figure 3(a)). The microvascular distribution pattern in the cat testes appears to be largely determined by the organ histology and especially the layout of the seminiferous tubules; therefore, 2nd-order arterioles, pre-capillary, as well as the capillaries (10–4 *μ*m), and postcapillary venules (10–30 *μ*m) that follow have almost a well defined hexagonal pattern of vessels surrounding the intertubular and peritubular patterns seminiferous tubules (Figure 3(b), 8(a), and 8(b)). Inside seminiferous tubule one can observe spermatogonia cells at different stages of maturation (Figure 9). Vascular features important in redirecting the blood flow and the amount of flow can be also observed in testis microvascularization, such as ostial valvulae (Figure 4) and intraarterial cushions (Figure 5).* Vasa vasorum* can be also observed (Figure 7).

**Figure 6:**
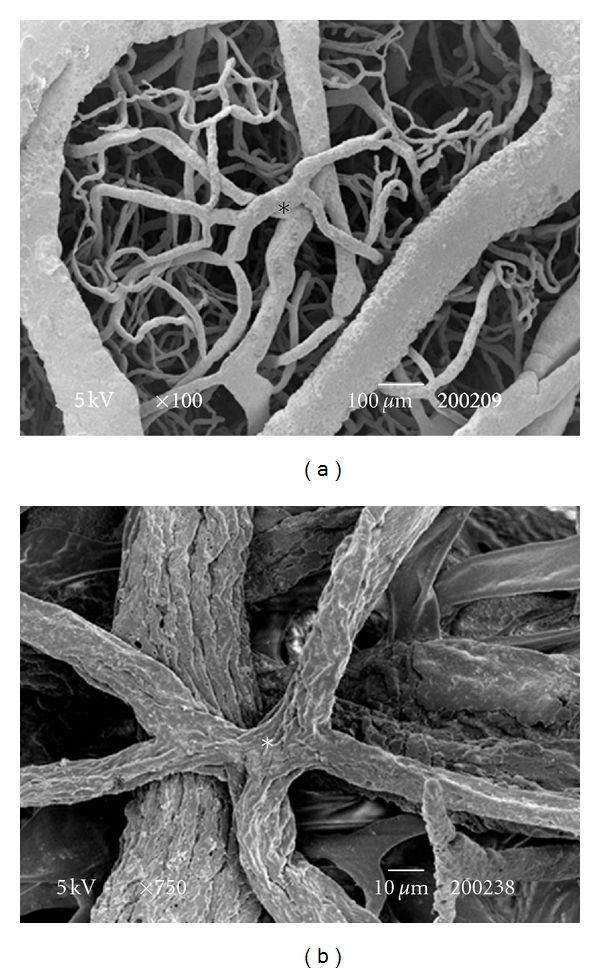
(a) SEM: peritubular vascularisation. Terminal arterioles often branch into vessels located at different segmental levels. This fact originates stelatae images (*). Bar = 100 *μ*m. (b) SEM: the same aspect. Bar = 10 *μ*m.

**Figure 7:**
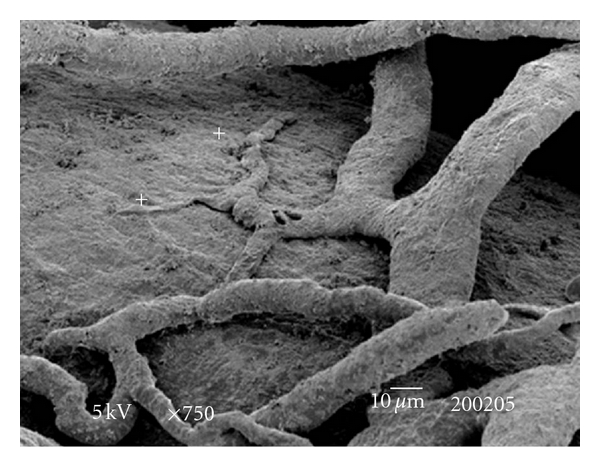
SEM: vasa vasorum (+) over a 2nd-order arteriola. Bar = 10 *μ*m.

**Figure 8:**
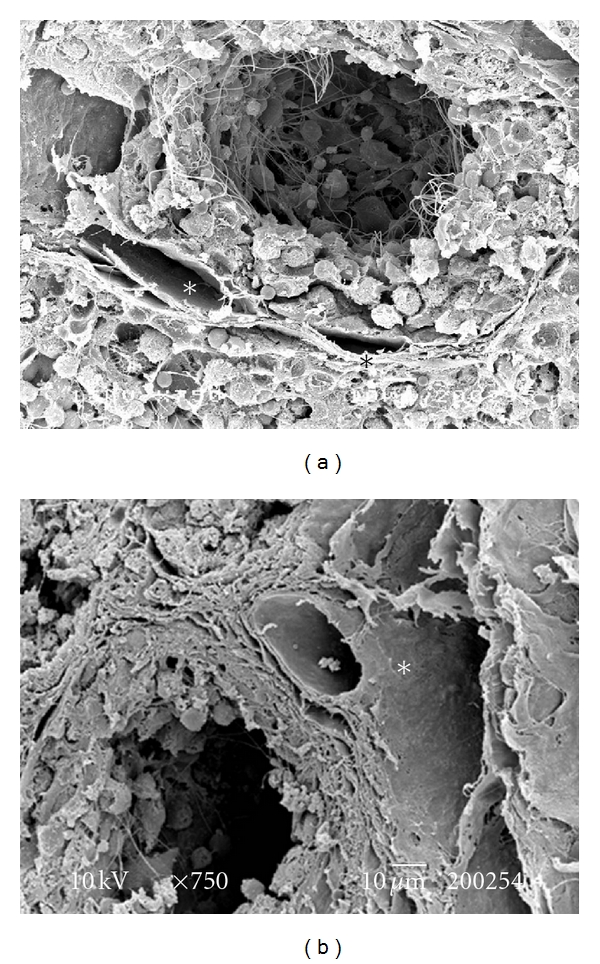
(a, b) SEM over intact tissue: seminiferous tubules showing different stages of maturation. Vessels (*) can be seen surrounding these tubules. Bar = 10 *μ*m.

**Figure 9:**
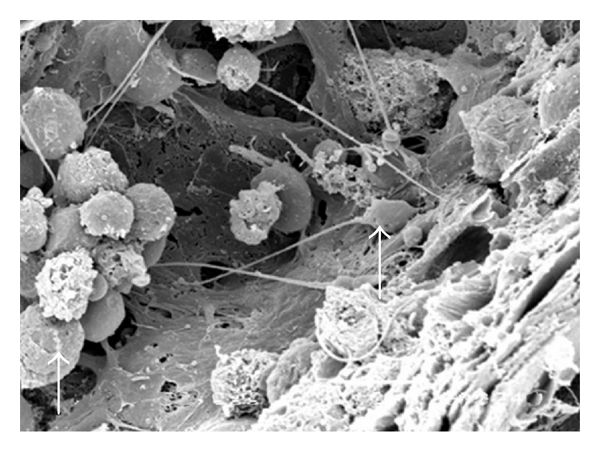
SEM over intact tissue: spermatogonias at different stages of maturation (arrows) inside a seminiferous tubule. Bar = 10 *μ*m.

Terminal arterioles often branch into vessels located at different segmental levels giving rise to an anastomotic net pattern (Figure 6(a)). As monopodical ramifications and dichotomic ramifications can be seen sprouting at the top of these vessels, one can observe *stelatae* ramifications (Figure 6(b)).

**Figure 10:**
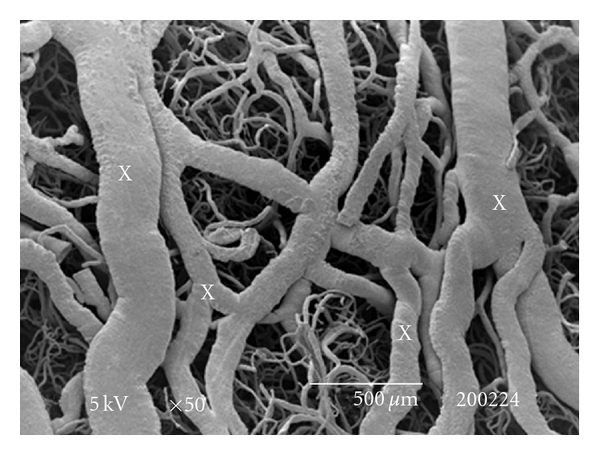
SEM: anastomosed network of flattened veins (X). Bar = 500 *μ*m.

2nd-order (30–100 microns) and 1st-order (100–200 microns) venules drain predominantly into an important anastomosed network of flattened veins, laid out in two superimposed subalbuginea vascular layers, which ultimately collect into a cranial plexus that form the pampiniform plexus (Figure 10).

**Figure 11:**
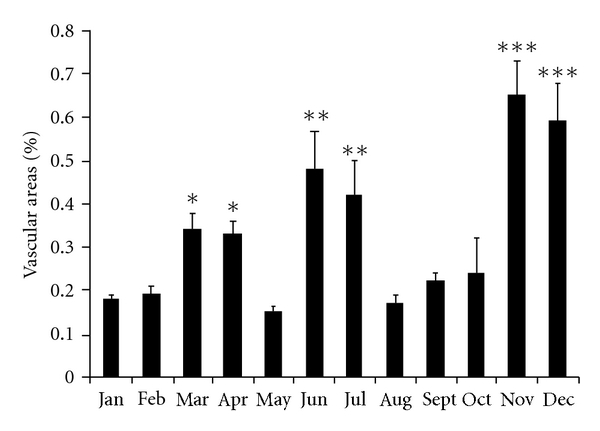
Vascular areas in testes throughout the year. A significant increase was observed in three different times of the year. A higher increase was observed in November and December when compared to the other months (***P < 0.001). Different symbols show significant differences: (**P < 0.01; *P < 0.05).

Our data points out an increase in microvascular areas in three different times of the year: March and April (*P* < 0.05); June and July (*P* < 0.01); November and December (*P* < 0.001), when compared to other months. In fact, it was in November and December that cat testes presented the highest microvascular areas when compared to the remaining months (Figure 11).

**Figure 12:**
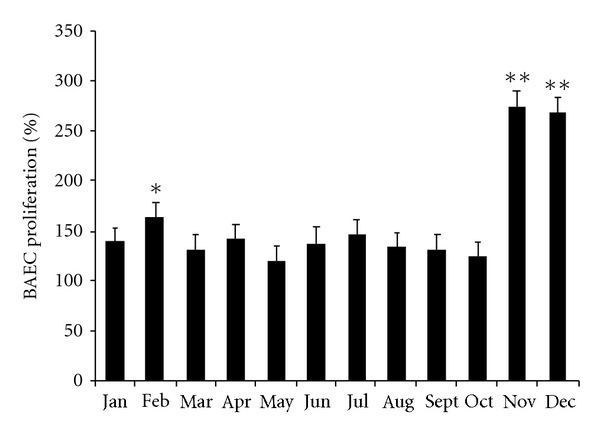
BAEC mitogenesis capacity of conditioned media by testes collected throughout the year. Different symbols show significant differences with other months (*P < 0.05; **P < 0.001).

It was also observed that cat testes tissue stimulates *in vitro* angiogenic activity differently. All testicular tissue showed the capability to increase BAEC proliferation, when compared to negative control throughout the study. In addition, there was an increase in BAEC mitogenic activity in February (*P* < 0.05) and November and December (*P* < 0.001), when compared to the other months (Figure 12).

**Figure 13:**
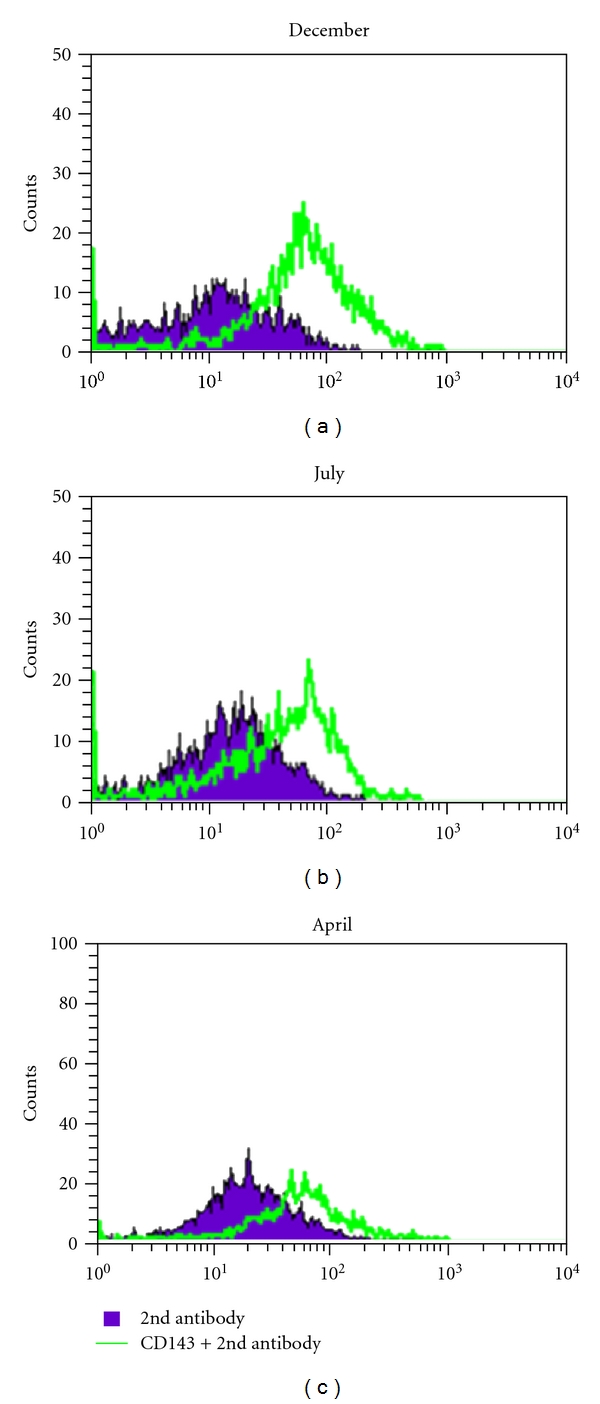
Examples of histograms showing the expression of CD143 in testes in different months of the year.

**Figure 14:**
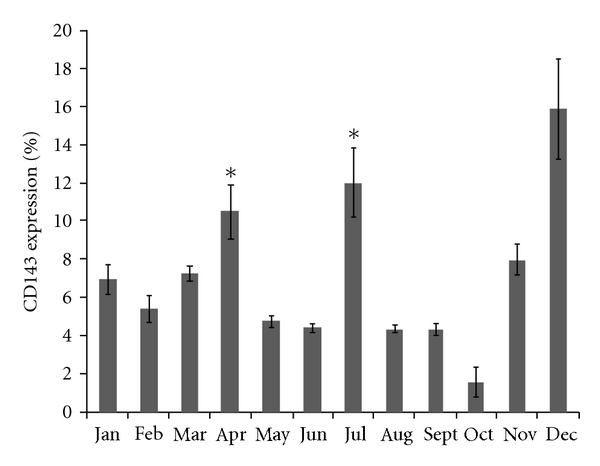
CD143 expression in the supernatant of ground testes along an entire year. An increase in CD143 expression was observed in December (*P* < 0.001) although smaller peaks were also observed in April and July (*P* < 0.01).

Considering flow cytometry studies regarding testicular endothelial cell evaluation, it was observed a 20 to 30% increase in CD143 expression in December (*P* < 0.001), although smaller peaks were present also in April and July (*P* < 0.01). The major immunophenotypic features are illustrated in Figures 13-14.

## 4. Discussion

The formation of new blood vessels from a preexisting vasculature, known as angiogenesis, is a quite infrequent process in adult mammals as a physiologic process. Both angiogenic and antiangiogenic factors play a regulatory role on angiogenesis [7, 31]. In the female reproductive tract during the ovarian/uterine cycle it occurs as physiologic changes [10–13], as well as in the male reproductive tract during gonadal recrudescence in seasonal breeders [14, 24, 32, 33].

It is known that testicular arteries of many animals (humans, mice, and rats) run from the abdominal aorta to the testes showing various configurations (straight, spiral, meandering, or coiled forms). Each species exhibits a specific pattern that may play several roles in protection of normal spermatogenesis, such as allowing wide mobility of the testes on physical attack, heat loss throughout the pampiniform plexus [34].

When evaluating the microvascular arrangement of arterial and venous vessels of cat testes, our data highlight the presence of intertubular and peritubular patterns found in precapillary or terminal arterioles, as well as in capillaries and postcapillary venules which seem to be similar to the patterns found in other species [35, 36], being the angioarchitecture largely determined by the testicular histology and especially the layout of the seminiferous tubules. Besides, venous organization in the cat testes might contribute for an efficient reduction in blood temperature within the testis, as second- and first-order venules which drain predominantly into an anastomosed network of flattened veins, laid out in two superimposed subalbuginea vascular layers that ultimately collect into a cranial plexus that form the pampiniform plexus. This outstanding venous plexiform apparatus, covering the cranial pole of the gonad seems to be particularly well suited to perform an important role in the thermoregulatory mechanisms of the testis, as mentioned before for bull [37], dolphin [38], and human [39].

Seasonal reproduction is mainly under photoperiodic control and is common among mammals at all latitudes. Photoperiod (day length) is a cue for temporal information in order to initiate and terminate seasonally appropriate morphological, physiological, and behavioural modifications that maximize survival as well as reproductive success [24]. This tool allows organisms to essentially track time-of-year and to anticipate relatively predictable annual variations in important environmental parameters [40, 41]. Photoperiod information obtained by the retina is transduced into a physiological signal via the pineal hormone, which secretes melatonin [40]. The molecular mechanisms at the hypothalamo-hypophysial level are involved in the secretion of melatonin at night which subsequently influences the gonadal axis in mammals [42]. Variations in response to photoperiod are seen not only among species but also between breeding populations within a species and between individuals within single-breeding populations [40]. This appears to be the result of differences in responsiveness to photoperiod or melatonin target sites responsiveness [40]. The molecular mechanisms that regulate vascular development and regression in response to environmental status are a subject with a lot of gaps in our knowledge. Seasonal changes in testicular capillary blood flow [43] and volume and density of testicular microvasculature [14] have been reported in several species that breed seasonally. It has also been pointed out that regression and regrowth of the white-footed mouse (*Peromyscus leucopus*) testes positively correlates with vascular endothelial growth factor (VEGF) protein expression [23]. Nevertheless, controversial studies in the White-Footed mouse do not support the idea that melatonin helps to regulate seasonal reproduction by acting in the testes to inhibit steroidogenesis [44]. Genetic differences have been pointed out in some species on both the degree and duration of reproductive quiescence [45]. Our data show that cat testicular tissue collected from different periods of the year was able to show angiogenic activity. The fact that the highest peak of BAEC mitogenesis in the testes occurred in November and December, and the greatest testicular vascular area was found in November and December as well, clearly points out that at this time of the year physiologic events are involved in the vascular growth of testes. Besides, in the same photoperiod window, a significant increase in CD143 expression in cat testes, which corresponds to a rise in endothelial cell proliferation (20 to 30% of total cell number), also occurred in December, which may indicate a stimulation in angiogenesis/vascular proliferation. This increase in angiogenic activity might be ascribed to testes increase in angiogenic/mitogenic factors, such as vascular endothelial growth factor (VEGF), angiopoietins (Angs), basic fibroblast growth factor (bFGF), and epidermal growth factor (EGF), considered to be crucial for new blood vessel growth [46–49]. Nevertheles, this raise in angiogenic activity in cats testes in Nov/Dec months might not only be due to increased angiogenic factors, but also to a reduction in the production of antiangiogenic factors, such as angiostatin, endostatin [8, 50], thrombospondins [51], and platelet factor 4- PF-4 [52]. In fact, an increase in VEGF expression in rat testes stimulated vascular endothelial cells and germ cells proliferation [53], while its inhibition was responsible for a reduction on *in vitro *testes vascular density [54]. Besides, gonadotrophins might also act directly on testis endothelial cells as tissue-specific angiogenic factors by modulating a more favorable vascular supply [55]. Since VEGF has been shown to stimulate testosterone release by rat Leydig cells in a dose dependent fashion [56], and testicular germ cell survival and sperm production in bulls [57], the present increase in vascularization might be related to an increase in testosterone secretion needed for cat reproduction function.

Research models that account for photoperiodic time measurement by a circadian mechanism postulate that the timing of light exposure, rather than the total amount of light, is critical to the pineal gland perception of day length and that might explain sudden modifications, with gaps of few months in testes activity. The present study points out three peaks considering vascular areas, namely, March and April, June and July, and November and December. Crossing this information with results obtained on mitogenesis assays and CD143 expression, it can be observed that December is committed to testes changes as it was demonstrated an increase in BAEC mitogenesis and an increase in CD143 expression also in this month.

Although a clear time-window can be related to November and December, a similar pattern can be observed in March-April and June-July, although with lower peaks. The increase in BAEC mitogenesis in February might represent an intermediate physiological state such as the ones observed between peripubertal and active adult testes [58].

Angiogenesis modifications might relate to function since testicular vasculature is unique in several ways. This is due to the presence of unfenestrated endothelial cells in the testis, which show about 10-fold higher proliferation rate when compared to other organs [59]. Endothelial cell mitogenesis, blood flow, and vascular permeability in testis could be further increased by gonadotrophin stimulation of Leydig cells [59–62]. As changes in angiogenesis may play a role on vascular growth and regression of the testes during the breeding and nonbreeding season in the male cat respectively, altogether, these data suggest that testicular vascularisation in cats appears to be predominantly increased in three photoperiod windows of time, November/December, March/April, and June/July. These findings are in accordance with the fact that most queens give birth at the beginning of the year, between May and July and in September (65–67 days of gestation). These windows of seasonality might be the response of circadian oscillators to the timing of light exposure, rather than the total amount of light. This mechanism might be involved in changes in testicular vascular pattern in the domestic cats that might influence their reproductive performance in the northern hemisphere at this range of latitude (38°43′N).
